# Under-diagnosis of SARS-CoV-2 infections among children aged 0–15 years, a nationwide seroprevalence study, Israel, January 2020 to March 2021

**DOI:** 10.2807/1560-7917.ES.2021.26.48.2101040

**Published:** 2021-12-02

**Authors:** Victoria Indenbaum, Yaniv Lustig, Ella Mendelson, Yael Hershkovitz, Aharona Glatman-Freedman, Lital Keinan-Boker, Ravit Bassal

**Affiliations:** 1 Central Virology Laboratory, Ministry of Health, Ramat-Gan, Israel; 2 Department of Epidemiology and Preventive Medicine, School of Public Health, Sackler Faculty of Medicine, Tel Aviv University, Tel Aviv, Israel; 3 Israel Center for Disease Control, Ministry of Health, Sheba Medical Center, Ramat-Gan, Israel; 4 School of Public Health, University of Haifa, Haifa, Israel

**Keywords:** SARS-CoV-2, COVID-19, seroprevalence study, Pfizer-BioNTech vaccine

## Abstract

Until recently, children and adolescents were not eligible for COVID-19 vaccination. They may have been a considerable source of SARS-CoV-2 spread. We evaluated SARS-CoV-2 IgG antibody seroprevalence in Israeli children aged 0–15 years from January 2020 to March 2021. Seropositivity was 1.8–5.5 times higher than COVID-19 incidence rates based on PCR testing. We found that SARS-CoV-2 infection among children is more prevalent than previously thought and emphasise the importance of seroprevalence studies to accurately estimate exposure.

The coronavirus disease (COVID-19) pandemic has spread globally since December 2019. Despite global evidence that children infected with severe acute respiratory syndrome coronavirus 2 (SARS-CoV-2) are more likely to manifest mild symptoms and are at a lower risk of developing severe respiratory disease, there is growing evidence of post COVID-19 condition among children and adolescents [1,2]. Available data on the transmissibility of SARS-CoV-2 among children are contradictory [3]. Moreover, the substantial percentage of asymptomatic infections makes it difficult to accurately estimate the incidence of SARS-CoV-2 infections among children and adolescents [4].

We present results from a nationwide serological study of children aged 0–15 years in Israel. Our aim was to estimate seropositivity during the COVID-19 pandemic, to evaluate the association between the reported confirmed cases and seropositivity rate and to identify the children's odds for SARS-CoV-2 exposure.

## COVID-19 vaccination in 12−16-year-olds

The rollout of COVID-19 vaccination among adolescents in Israel started in June 2021. By 3 November 2021, 57% of adolescents (aged 12–16 years) in Israel had received their first vaccine dose and 46% had received their second vaccine dose [5]. As at 22 November 2021, the COVID-19 vaccine has been approved for 5–11-year-olds in Israel. As a result, a considerable number of children under the age of 16 years in Israel are thought to be susceptible to SARS-CoV-2 infection.

## Seroprevalence study

We studied 2,765 serum samples collected between 1 January 2020 and 31 March 2021 from children aged 0–15 years, with a median age of 8.9 years (interquartile range: 4.1–13.1). Samples were obtained anonymously from individuals who performed routine or diagnostic blood tests in one of five laboratories located throughout Israel by the Israel National Serum Bank (INSB) [6]. None of the children had received a COVID-19 vaccine. Demographic data of the study cohort are presented in Table 1.

**Table 1:** Demographic data and seropositivity of children aged 0–15 years, Israel, January 2020–March 2021 (n = 2,765)

Characteristics	Study participants			Seropositivity			
	n	%		n	%	95% CI	p value
Total n	2,765	100		156	5.6	4.8–6.6	NA
Age							
Median age (IQR)	8.93 (4.1–13.1)			NA			
Age group							
< 6 months	80	2.9		6	7.5	2.8–15.6	0.406
6–12 months	63	2.3		1	1.6	0.0–8.5	
1–4 years	729	26.4		34	4.7	3.2–6.5	
5–9 years	685	24.8		38	5.6	4.0–7.5	
10–11 years	314	11.4		19	6.0	3.7–9.3	
12–15 years	894	32.3		58	6.5	5.0–8.3	
Sex							
Male	1,436	51.9		82	5.7	4.6–7.0	0.871
Female	1,329	48.1		74	5.6	4.4–6.9	
Ethnicity							
Jewish and others^a^	1,960		72.5	104	5.3	4.4–6.4	0.091
Arab	742		27.5	52	7.0	5.4–9.1	
District							
Jerusalem	191		6.9	21	11.0	6.9–16.3	0.001
North	489		17.7	26	5.3	3.5–7.7	
Haifa	336		12.2	16	4.8	2.8–7.6	
Judea and Samaria	170		6.2	16	9.4	5.5–14.8	
Central	599		21.7	32	5.3	3.7–7.5	
Tel Aviv	225		8.1	17	7.6	4.5–11.8	
South	755		27.3	28	3.7	2.5–5.3	
SES							
Median SES (range)	5 (1–10)			NA			
High SES (6–10)	899		32.5	30	3.3	2.3–4.7	< 0.001
Low SES (1–5)	1,475		53.3	107	7.2	6.0–8.7	
Unknown SES	391		14.1	19	4.9	3.0–7.5	

CI: confidence interval; IQR: interquartile range; NA: not applicable; SES: socio-economic status.

^a^ Brazilian, Belarusian, Chilean, Circassian, Ethiopian, Finnish, Hungarian, Indian, Russian, Ukrainian (n = 40 samples).

The samples were collected from all seven districts of Israel (Jerusalem, North, Haifa, Judea and Samaria, Central, Tel Aviv and South) and represented children from different regions, socio-economic status and population groups. The data on socio-economic status were allocated to each participant based on their address of residence using the socio-economic residential classification and was divided into low (clusters 1–5) and high (clusters 6–10) [7].

All samples were initially screened for SARS-CoV-2 specific IgG antibodies using an in-house ELISA based on the receptor-binding domain assay [8]. In this assay, a sample-to cut-off ratio (S/CO) equal to or above 1.1 is considered to be positive and below, negative, with 88% sensitivity and 98% specificity [8,9]. To increase sensitivity and specificity, we tested 76 samples with a S/CO below 1.1 using a SARS-CoV-2 pseudo-virus neutralisation assay (NA) [10]. While all 47 samples below 0.8 S/CO were negative, seven samples with a S/CO of 0.8–1.1 were positive, suggesting that there was no reason to test samples below 0.8 with the confirmatory NA. Therefore, all samples with a S/CO of 0.8 or above were tested using NA. Levels of 16 or above were considered neutralising and thus, seropositive. Levels below 16 were considered not to be neutralising and hence, negative.

## Seropositivity rates analysis

The number, percentage and 95% confidence interval (CI) of seropositive individuals in general and by certain characteristics are presented in Table 1. Overall, 5.6% (95% CI: 4.8–6.6; n = 156) of samples were seropositive. No statistically significant difference was found among different age and population groups. Seropositivity rate was 11% (95% CI: 6.9–16.3) in the Jerusalem district, significantly higher than in other districts. Lower socio-economic status was associated with significantly higher seropositivity; 7.2% (95% CI: 6.0–8.7) of low socio-economic status children and 3.3% (95% CI: 2.3–4.7) of high socio-economic status children were seropositive.

The Figure demonstrates the percentage of seropositivity over time during the study observation period.

**Figure:**
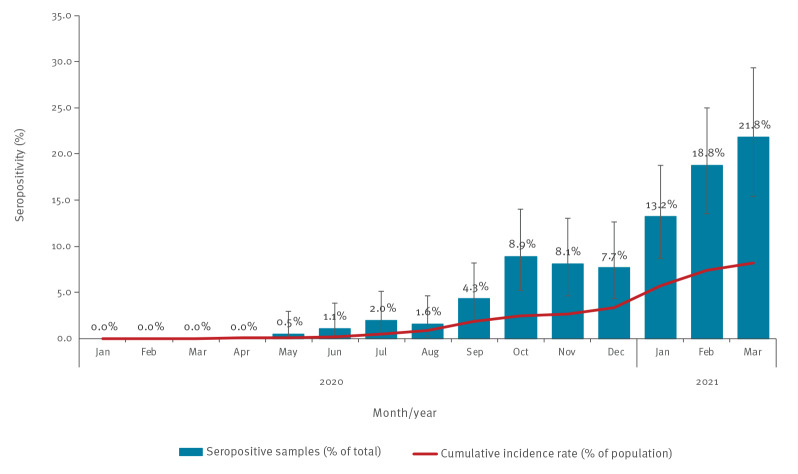
SARS-CoV-2 seropositivity and cumulative incidence rate in children aged 0–15 years, Israel, January 2020–March 2021

Percentages of SARS-CoV-2 positive serum samples (blue boxes) and cumulative SARS-CoV-2 incidence rate in children (red line) from January 2020 to March 2021. Bars represent 95% confidence intervals.

A positive association between seropositivity and time was observed. Specifically, no seropositive samples were observed between January 2020 and March 2020; the percentage of seropositive samples started to rise in May 2020 and reached 21.8% (95% CI: 15.4–29.3) in March 2021, the last month of the study period. We compared the cumulative incidence rate of SARS-CoV-2 positive children confirmed by RT-PCR as documented in the national SARS-CoV-2 repository [5] and the percentage of seropositive samples detected during the study period (Figure). From June 2020 to March 2021, seropositivity was between 1.8 and 5.5 times higher than the cumulative SARS-CoV-2 incidence rate.

## Association between population characteristics and SARS-CoV-2 seropositivity

The associations between seropositivity and risk factors as determined by univariate analysis were examined using logistic regression to calculate p values, odds ratios (OR) and 95% CI. The results presented in Table 2 show that children living in the Jerusalem district had higher odds (OR: 2.2; 95% CI: 1.2–4.0) of being SARS-CoV-2-seropositive than children living in the Northern district, and children of low socio-economic status had higher odds (OR: 2.3; 95% CI: 1.5–3.4) of being SARS-CoV-2-seropositive than children from high socio-economic status. There were no significant differences in seropositivity between different age groups, sex and ethnic groups.

**Table 2:** Association between population characteristics and SARS-CoV-2 seropositivity, Israel, January 2020–March 2021 (n = 156)

Characteristics	Seropositivity	
	OR (95% CI)	p value
Age group		
< 6 months	5.0 (0.6–42.9)	0.140
6–12 months	Ref	NA
1–4 years	3.0 (0.4–22.5)	0.278
5–9 years	3.6 (0.5–27.0)	0.206
10–11 years	4.0 (0.5–30.4)	0.181
12–15 years	4.3 (0.6–31.6)	0.152
Sex		
Male	1.0 (0.7–1.4)	0.871
Female	Ref	NA
Ethinicity		
Jewish and others^a^	Ref	NA
Arab	1.3 (1.0–1.9)	0.091
District		
Jerusalem	2.2 (1.2–4.0)	0.010
North	Ref	NA
Haifa	0.9 (0.5–1.7)	0.722
Judea and Samaria	1.8 (1.0–3.5)	0.063
Central	1.0 (0.6–1.7)	0.985
Tel Aviv	1.5 (0.8–2.7)	0.245
South	0.7 (0.4–1.2)	0.176
SES		
Median SES (range)	5 (1–10)	NA
High SES (6–10)	Ref	0.001
Low SES (1–5)	2.3 (1.5–3.4)	

CI: confidence interval; NA: not applicable; OR: odds ratios; Ref: reference; SARS-CoV-2: severe acute respiratory syndrome coronavirus 2; SES: socio-economic status.

^a^ Brazilian, Belarusian, Chilean, Circassian, Ethiopian, Finnish, Hungarian, Indian, Russian, Ukrainian (n = 40 samples).

### Ethical statement

These data were collected as part of surveillance by the Israel Ministry of Health and thus require no ethical clearance. Additionally, the serosurvey is based on the INSB samples which are anonymous.

## Discussion

In May 2021, the United States (US) Food and Drug Administration (FDA) and the European Medicines Agency (EMA) approved the use of the BNT162b2 mRNA COVID-19 vaccine (Pfizer, New York, US and BioNTech, Mainz, Germany) for children aged 12–15 years [11,12]. On 29 October 2021, the authorisation was expanded by the FDA to include younger children aged 5–11 years with a smaller vaccine dose [13]. The EMA also recommended an extension of indication for the BNT162b2 mRNA COVID-19 vaccine to include use in children aged 5–11 years on 25 November 2021 [14]. However, to date, vaccine uptake by children and adolescents is progressing slowly worldwide. As a result, people under the age of 16 years remain susceptible to SARS-CoV-2 infection and a source for continued circulation. Therefore, it is critical to assess the magnitude of undiagnosed infections in this age group.

Our results suggest that, in the general population, most infections in individuals under 16 years of age are undiagnosed. As at May 2020, we observed an increase in the percentage of seropositive samples, from 0.5% in May 2020 to 21.8% in March 2021. However, the percentage of PCR-confirmed infections during the same time period was substantially smaller. At the beginning of the pandemic during the first wave (May–June 2020) the seropositivity percentage was 5–5.5 times higher than the percentage of PCR-confirmed cases. This probably resulted from low availability and limitations imposed on PCR testing and monitoring of suspected cases at the beginning of the pandemic. Later on, following a considerable improvement in the availability of PCR testing and monitoring of suspected cases, the difference between the two measurements decreased. Starting in September 2020, a 2–3-fold difference between COVID-19 incidence and seropositivity was observed and was sustained until March 2021. Our observations are in line with accumulative data demonstrating that most children infected with SARS-CoV-2 experience clinically mild disease or remain asymptomatically infected [15,16].

The finding that children living in the Jerusalem district and those with a low socio-economic status had twofold higher odds of being SARS-CoV-2 seropositive as compared with children living in the Northern district and those with a high socio-economic status, respectively, is consistent with population differences found during the pandemic in Israel. Specifically, more than 30% of the Jerusalem population belongs to the ultra-Orthodox community, which was severely affected by the COVID-19 pandemic [17]. This community consists of many large families living in small apartments, resulting in a crowded environment. Most of them have a low socio-economic status. Structural, religious and social-ideological factors directly or indirectly influenced the high SARS-CoV-2 infection rates among the ultra-Orthodox population in Israel [17].

Although this cohort of the Israeli population was limited in number, through this study we found that more than 20% of children aged 0–15 years in Israel had been infected with SARS-CoV-2 as at March 2021. The use of cumulative COVID-19 incidence over time allowed us to determine that overall, at least 50% of SARS-CoV-2 infections in this age group, were undiagnosed between September 2020 and March 2021 based on the difference observed between cumulative incidence and seropositivity. While our results may suggest that natural infection is more prevalent than anticipated asymptomatic infection, especially in younger ages, might result in low and unsustained antibody levels [18].

### Conclusion

This study emphasises the importance of regular seroprevalence studies to estimate the population's immunity to SARS-CoV-2. Such studies enable characterisation of the fraction of the population that still needs to be protected and can facilitate efforts to reach these individuals. Future studies should investigate the impact of the Delta or other variants of concern on asymptomatic vs symptomatic infections in children and adolescents. Serological studies can also serve as a tool in vaccination programmes planned by countries.
